# Rituximab in combination with gemcitabine plus cisplatin in patients with recurrent and metastatic head and neck squamous cell carcinoma: a phase I trial

**DOI:** 10.1186/s12885-022-09258-0

**Published:** 2022-02-15

**Authors:** Ching-Yun Hsieh, Ming-Yu Lien, Chen-Yuan Lin, Wen-Jyi Lo, Chung-Hung Hua, Wei-Chao Chang, Chang-Fang Chiu, Ching-Chan Lin

**Affiliations:** 1 Division of Hematology and Oncology, Department of Internal Medicine, China Medical University Hospital, China Medical University, 2 Yude Rd, North District, Taichung, 404 Taiwan; 2 School of Medicine, China Medical University, Taichung, Taiwan; 3 School of Pharmacy and Graduate Institute, China Medical University, Taichung, Taiwan; 4 Department of Otorhinolaryngology, China Medical University Hospital, China Medical University, Taichung, 404 Taiwan; 5 Graduate Institute of Biomedical Sciences and Center for Molecular Medicine, China Medical University, Taichung, Taiwan

**Keywords:** Head and neck squamous-cell carcinoma, Rituximab, Chemotherapy, Anti-CD20 antibody

## Abstract

**Background:**

The treatment of recurrent or metastatic head and neck squamous-cell carcinoma (R/M HNSCC) remains challenging. Preclinical studies revealed that B cell depletion could modulate the microenvironment and overcome chemoresistance. We conducted a phase I study to evaluate the feasibility and safety of B cell depletion using the anti-CD20 antibody rituximab to treat HNSCC.

**Methods:**

Ten patients were enrolled in two protocols. The first four patients treated using protocol 1 received rituximab 1000 mg on days −14 and −7, followed by gemcitabine/cisplatin every 3 weeks, and rituximab was administered every 6 months thereafter. Because of disease hyperprogression, protocol 1 was amended to protocol 2, which consisted of the concomitant administration of rituximab 375 mg/m^2^ and gemcitabine/cisplatin every 3 weeks. Another six patients were enrolled and treated using protocol 2.

**Results:**

Three patients treated using protocol 1 exhibited rapid disease progression, and the remaining patient could not undergo evaluation after rituximab treatment. Conversely, no unpredicted harm was observed in the six patients treated using protocol 2. Among these patients, one achieved complete response, and two had partial responses. The disease-free durations in these patients were 7.0, 6.2, and 7.1 months, respectively. Immune cell analysis revealed a higher ratio of cytotoxic T cells to regulatory T cells in responders than in non-responders.

**Conclusions:**

B cell depletion using rituximab alone in patients with HNSCC can cause hyperprogressive disease. Contrarily, the co-administration of rituximab and cisplatin/gemcitabine was feasible and safe.

**Trial registration:**

ClinicalTrials.gov Identifier: NCT04361409, 24 April 2020, retrospectively registered

## Background

Recurrent or metastatic squamous-cell carcinoma of the head and neck (R/M HNSCC) has a dismal prognosis [1]. Systemic therapies such as chemotherapy or targeted therapy against EGFR have been the mainstay of palliation [2], but long-term disease control is difficult. The development of programmed death 1 (PD-1) immune checkpoint inhibitors changed the treatment of HNSCC. Pembrolizumab produced a durable response rate and better overall survival than cetuximab plus platinum chemotherapy [3]. However, less than 20% of patients respond to this treatment, and the median progression-free survival is approximately 5 months. Therefore, novel therapies are urgently required for patients with R/M HNSCC.

The importance of B cells in the tumor microenvironment (TME) has been increasingly investigated [4]. An animal study demonstrated that CD20+ B cells are responsible for chemoresistance to platinum agents and taxanes in HNSCC [5]. B cell depletion reduced chemotherapy resistance of squamous-cell carcinoma in mouse models [5]. Clinically, the anti-CD20 antibody rituximab has been effectively used to directly target CD20+ hematologic malignancies [6, 7]. A case series identified rituximab as a therapeutic option for advanced malignant melanoma [8], and a study in colon cancer demonstrated that rituximab reduced B cell counts and exerted a considerable clinical effect [9].

Gemcitabine produces a modest response rate (0–13%) as a single agent in patients with R/M HNSCC [10]. Synergistic activity between gemcitabine and cisplatin was observed in preclinical and clinical data [11]. Gemcitabine more potently inhibits B cell proliferation than T cell proliferation [12]. Therefore, gemcitabine may be a practical treatment option in conjunction with a variety of immunotherapies for B cell depletion.

As B cell depletion was hypothesized to improve chemotherapeutic efficacy in HNSCC in preclinical studies, we conducted a pilot study to investigate the feasibility of rituximab combined with gemcitabine/cisplatin in R/M HNSCC.

## Methods

### Patients and study design

This open-label phase I pilot study (ClinicalTrials.gov Identifier: NCT04361409, 24/April/2020) examined the feasibility of the combination of rituximab and gemcitabine plus cisplatin in patients with R/M HNSCC. Eligible patients were more than 20 years old with histologically confirmed HNSCC. All patients had surgically unresectable lesions that progressed on standard treatment and no available effective treatment option at the time of study enrollment. All patients had measurable diseases as defined as the presence of at least one lesion as being ≥10 mm in at least one dimension measured with conventional computed tomography (CT) or ≥10 mm in at least one dimension measured with spiral CT scan or magnetic resonance imaging (MRI). The other main eligibility criteria included an Eastern Cooperative Oncology Group performance status of 0–2, adequate renal function (creatinine clearance ≤ 60 ml/min), and adequate hematologic and liver function. Key exclusion criteria were brain metastases, spinal cord compression, and only bone metastasis.

The other main eligibility criteria included an Eastern Cooperative Oncology Group performance status of 0–2 and adequate organ function. Key exclusion criteria were brain metastases, spinal cord compression, and only bone metastasis.

The study enrolled patients with R/M HNSCC in China Medical University Hospital, Taiwan. The study was conducted in accordance with the World Medical Association Declaration of Helsinki (version 2002) and approved by the China Medical University Hospital Review Board. Patients provided IRB-approved, protocol-specific written informed consent prior to receiving study-specific treatment.

Initially, the protocol (protocol 1) was designed to administer rituximab to deplete B cells before the administration of chemotherapy. Intravenous rituximab 1000 mg intravenously was administered on days −14 and −7 and every 6 months thereafter. Chemotherapy featuring cisplatin (70 mg/m^2^ intravenously) on day 1 and gemcitabine (1000 mg/m^2^ intravenously) on days 1 and 8 was administered every 21 days. After four patients were enrolled into protocol 1, the protocol was amended to administer rituximab (375 mg/m^2^ intravenously) on Day 1, followed by cisplatin and gemcitabine using the previously mentioned doses and schedule (protocol 2). Six patients were treated using protocol 2. The protocol schemes are presented in Table 1.

**Table 1:** Protocol schemes

	Protocol 1 (*n* = 4)	Protocol 2 (*n* = 6)
Rituximab	1000 mg on days −14 and −7, followed by treatment every 6 months	375 mg/m^2^ on day 1 every 3 weeks
Cisplatin	70 mg/m^2^ on day 1 every 3 weeks	70 mg/m^2^ on day 1 every 3 weeks
Gemcitabine	1000 mg/m^2^ on days 1 and 8 every 3 weeks	1000 mg/m^2^ on days 1 and 8 every 3 weeks

### Endpoints and disease response

The primary objective was to determine the feasibility of the combination of rituximab and gemcitabine/cisplatin in patients with R/M HNSCC, whereas the secondary objectives included the response rate, progression-free survival (PFS), and toxicity.

The major objective measurement for feasibility was to identify unpredicted harm during the course of combination treatment. The two feasibility objectives of our pilot study were to assess (1) unpredicted harm during the course of rituximab administration and (2) compliance with the post-rituximab examination. The feasibility objectives for our study were considered successful if the following criteria were met: (1) 80% of patients who received rituximab experienced no unpredicted harm during the course of rituximab administration and (2) 80% of patients could undergo examination 1 week after rituximab administration. Unpredicted harm was defined as death within 1 month after rituximab administration, severe infection or grade 3 tumor bleeding within 1 month after rituximab administration, or patient refusal to complete the second cycle of rituximab therapy.

PFS was defined as the time from study registration to the first day of disease progression at any site or of death by any cause. The response assessment was performed using computed tomography (CT) at the end of treatment according to RECIST 1.1. CT was performed every 8–12 weeks until disease progression. Furthermore, toxicities were assessed at each weekly visit during treatment and at the end of treatment and recorded by the investigators using NCI-CTCAE version 4.03.

### Evaluation of tumor growth kinetics ratios (TGKRs)

To evaluate TGKRs, patients underwent pre-baseline, baseline, and post-treatment CT. The minimal interval between two CT scans was 14 days, and patients were required to start rituximab within 1 week after the baseline scan. Tumor growth kinetics before (TGK_pre_) and after (TGK_post_) rituximab were evaluated. TGK_pre_ was defined as the difference of the sum of the largest diameters of the target lesions per unit of time between pre-baseline and baseline imaging [13]. TGK_post_ was defined in the same manner between on-treatment and post-treatment imaging. TGKR was calculated using RECIST 1.1 as the ratio of TGK_post_ to TGK_pre_. Hyperprogressive disease (HPD) was defined as TGKR ≥ 2. Tumor growth acceleration was indicated by 2 > TGKR > 1, whereas 0 < TGKR < 1 indicated tumor deceleration. TGKR < 0 indicated tumor shrinkage [13].

### Immune cell evaluation

Peripheral blood was obtained from patients prior to treatment and on day 7 after the administration of rituximab. At least 5 × 10^5^ events per sample were acquired on a six-color flow cytometer (BD FACSCanto II). Immunophenotypes were analyzed using CD20-FITC, CD16-PE, CD14-PerCP, CD19-APC, CD45-APC-H7, CD3-PE-C7, CD4-APC, CD127-PerCP, CD25-FITC, and CD8-FITC. Data analysis was performed using Infinicyt software (Cytognos, Salamanca, Spain).

### Statistical analysis

Statistical analysis was performed using SPSS 26 (SPSS Inc.). Graphs were created using GraphPad Prism version 7 (GraphPad software Inc.). The Mann–Whitney U test was used to compare continuous variables in all comparisons. Statistical significance was defined as *p*-value < 0.05.

## Results

### General characteristics

Ten patients, all of whom were men, were enrolled in China Medical University Hospital, Taiwan between August 2013 and September 2016. The patients’ characteristics are presented in Table 2. The median patient age was 47.5 (range, 33–61). The site of disease was the hypopharynx–oropharynx in two patients, the buccal cavity in two patients, and the tongue in two patients. Four patients developed distant metastases, and six had localized progression. Two of ten patients had persistent disease (Patient No.5 and No.7) and another eight patients had local recurrence or metastatic recurrence (Patient No. 4, No.6, and No.9) after definitive treatment. All patients had the habits of betel quid chewing, cigarette smoking, and alcohol consumption. All patients were cisplatin-refractory, which means patients had persistent diseases during cisplatin administration or recurrent diseases within six months after cisplatin administration. Two patients (Patient No.5 and No.7) were refractory to cisplatin at locally advanced stage and another eight patients were refractory to cisplatin at relapse and metastatic stage. All patients except Patients No.7 and No.10 received cetuximab when disease recurrence or distant metastasis occurred. Five patients (Patient No. 1–5) experienced progression despite receiving a cetuximab-containing regimen. Nine patients were refractory to concurrent chemoradiotherapy (Patient No. 1–6 and 8–10). No patients had received immune checkpoint inhibitors at the time of study enrollment.

**Table 2:** Patients’ demographic data

Patient no.	Age (years)	Sex	Habit of betel quid chewing, cigarette smoking and alcohol consumption	Primary tumor site	Previous lines of treatment^a^	Recurrence type	Cisplatin refractory	Cetuximab exposure	Treatment schedule	Best response	PFS (months)
1	47	Male	Yes	Buccal cavity	3	local	Yes	Yes	R then CT	SD	6.3
2	38	Male	Yes	Buccal cavity	3	local	Yes	Yes	R then CT	PD	1.9
3	49	Male	Yes	Hypopharynx	3	local	Yes	Yes	R then CT	PD	1.1
4	49	Male	Yes	Hypopharynx	3	Local +distant	Yes	Yes	R then CT	PD	1.9
5	39	Male	Yes	Tongue	2	Local (persistent)	Yes	No	R plus CT	PR	7.0
6	58	Male	Yes	Hypopharynx	2	Distant	Yes	Yes	R plus CT	CR	6.2
7	61	Male	Yes	Hypopharynx	1	Distant (persistent)	Yes	No	R plus CT	PD	1.6
8	33	Male	Yes	Tongue	3	Local	Yes	Yes	R plus CT	PD	0.7
9	48	Male	Yes	Oropharynx	2	Distant	Yes	Yes	R plus CT	PR	7.1
10	45	Male	Yes	Oropharynx	2	Local	Yes	No	R plus CT	SD	3.0

*PFS* progression-free survival, *R* rituximab, *CT* chemotherapy, *SD* stable disease, *PD* progressive disease *PR* partial response, *CR* complete response

^a^ including definitive treatment for locally advanced diseases

### Feasibility evaluation

The first four patients (Patient No. 1–4) were treated using protocol 1. Three of these patients completed all evaluations after treatment, and the remaining patient (Patient No. 4) could not undergo CT because of upper airway compression secondary to disease progression. Patient No. 5 initially received rituximab 1000 mg on day −14. However, his tongue pain worsened, and an episode of tumor bleeding occurred 3 days later. After suspecting disease progression, salvage chemotherapy with gemcitabine and cisplatin was administered. The tumor bleeding subsided, and his tongue pain improved obviously. Following discussions with the local IRB, we amended the protocol to include the co-administration of rituximab with gemcitabine/cisplatin every 3 weeks (protocol 2). Finally, six patients, including Patient No.5, were treated using protocol 2. No unexpected adverse effects were observed in patients treated using protocol 2, and all patients could be evaluated 1 week after rituximab administration.

The other adverse events during treatment are presented in Table 3. Most patients had grade 1–2 side effects, but two patients each had grade 3–4 nausea and grade 3–4 oral mucositis. The grade 3–4 hematologic side effects included grade 3–4 neutropenia in three patients, grade 3–4 anemia in two patients, and febrile neutropenia in two patients.

**Table 3:** Adverse events graded using NCI-CTCAE version 4.03

NCI-CTCAE grade	Grade 1–2	Grade 3–4
During induction chemotherapy (*n* = 10)		
Anemia	5	1
Neutropenia	5	3
Thrombocytopenia	1	0
Alopecia	3	
Mucositis/stomatitis	3	1
Febrile neutropenia	2	
Nausea	5	2
Vomiting	1	0
Fatigue	7	1
Peripheral neuropathy	2	0

### Response evaluation

**Fig. 1:**
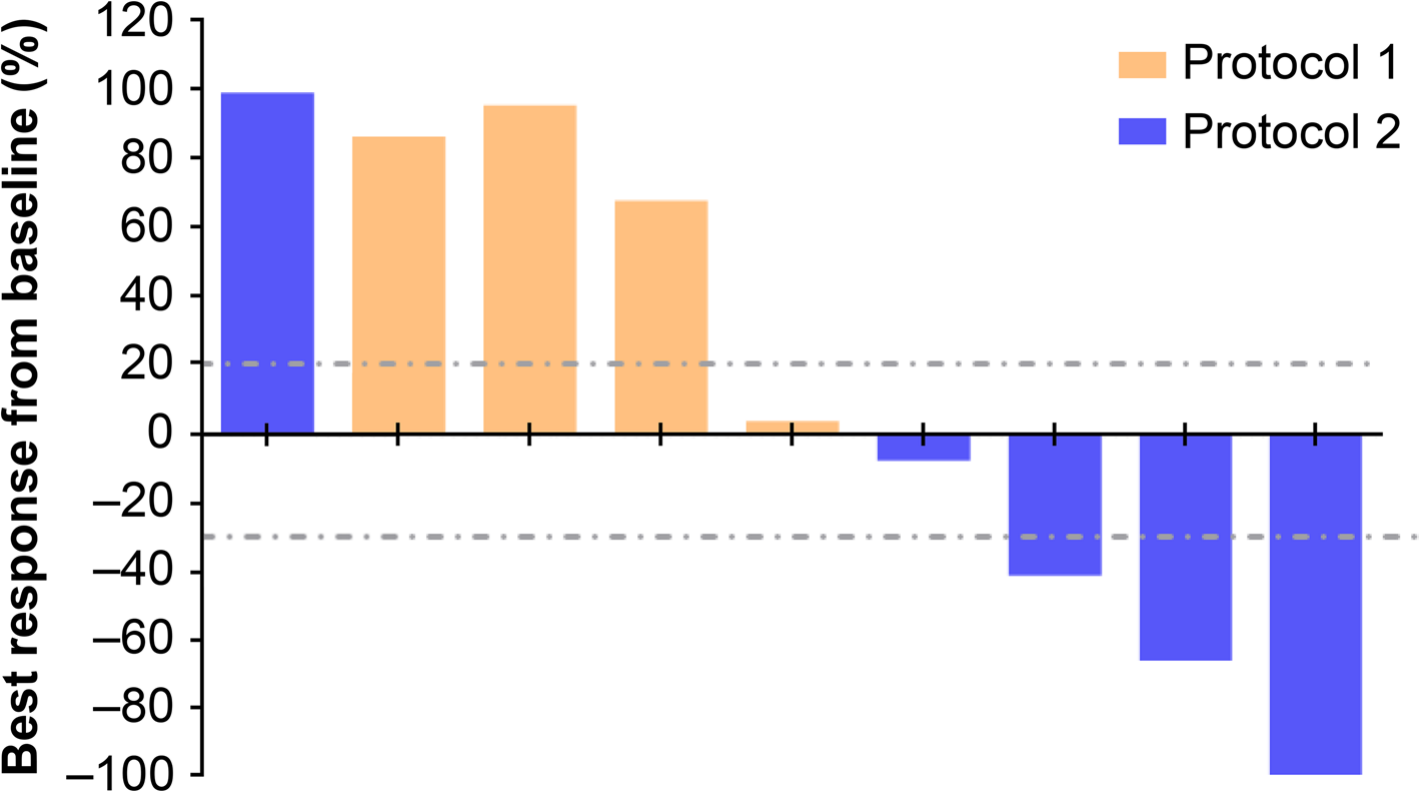
Waterfall plot of the best clinical response (RECIST 1.1) after treatment

**Fig. 2:**
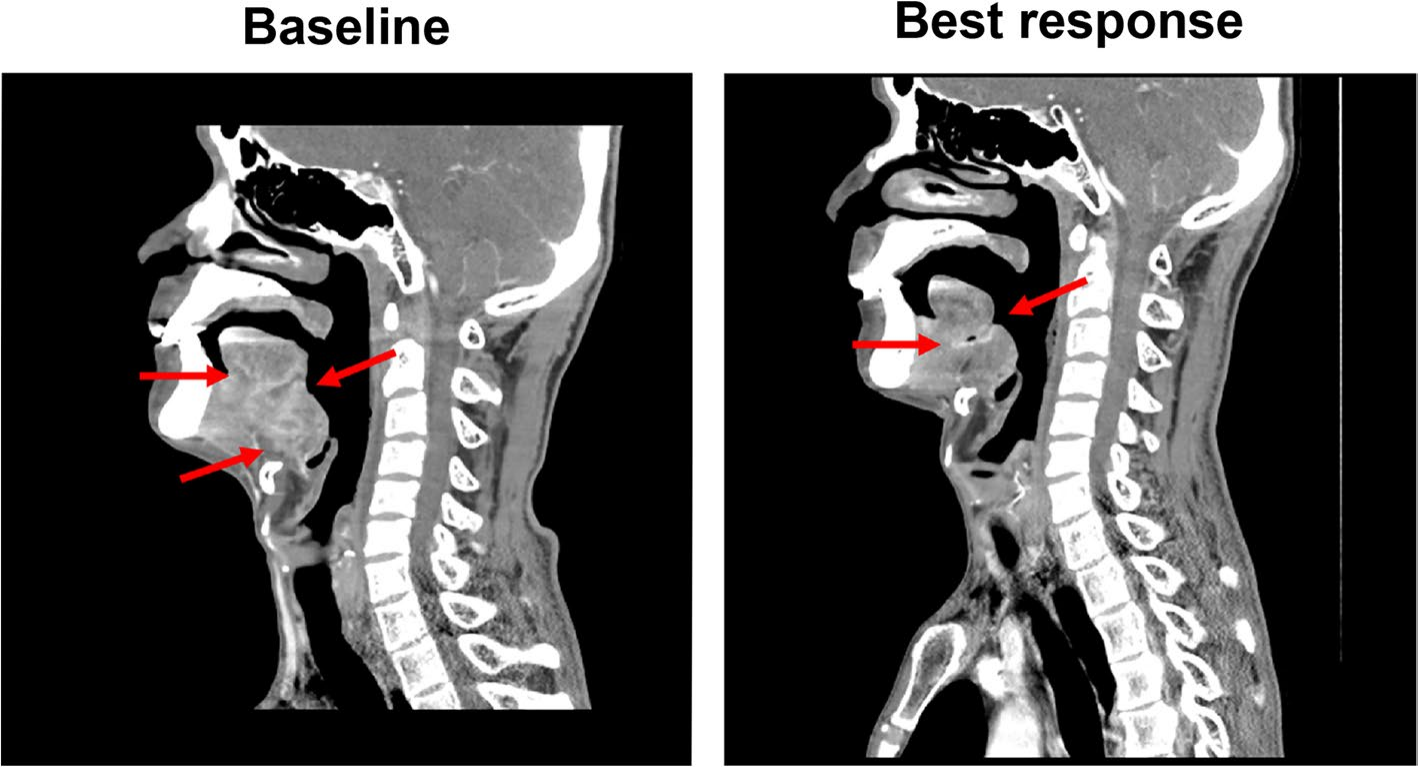
Computed tomography (CT) images of one responder. The patient had primary tongue cancer that was refractory to chemoradiotherapy using triweekly cisplatin 100 mg/m^2^. **a** Baseline CT at the sagittal plane (**b**) After 2 months, CT at the sagittal plane revealed a tumor shrinkage. Evaluation at axial planes by RECIST criteria revealed a partial response

The mean time elapsed from study enrollment to the administration of study drug was 2 weeks. In protocol 1, three patients experienced disease progression, and one patient had the best response of stable disease (Patient No. 1). Patient No.5, initially treated by protocol, had a partial response after receiving rituximab plus chemotherapy. In protocol 2, two patients responded to treatment, including one complete response (Patient No. 6) and one partial response (Patient No. 9). Two patients experienced disease progression, and the remaining patient had stable disease. Among the five patients experiencing clinical benefits (including complete responses, partial responses, and stable disease), the median duration of response was 6 months (range, 3–7.1). The waterflow plot of the patients’ best responses is presented in Fig. 1. The representative CT findings of one responder (Patient No. 5) before and after treatment are presented in Fig. 2.

**Fig. 3:**
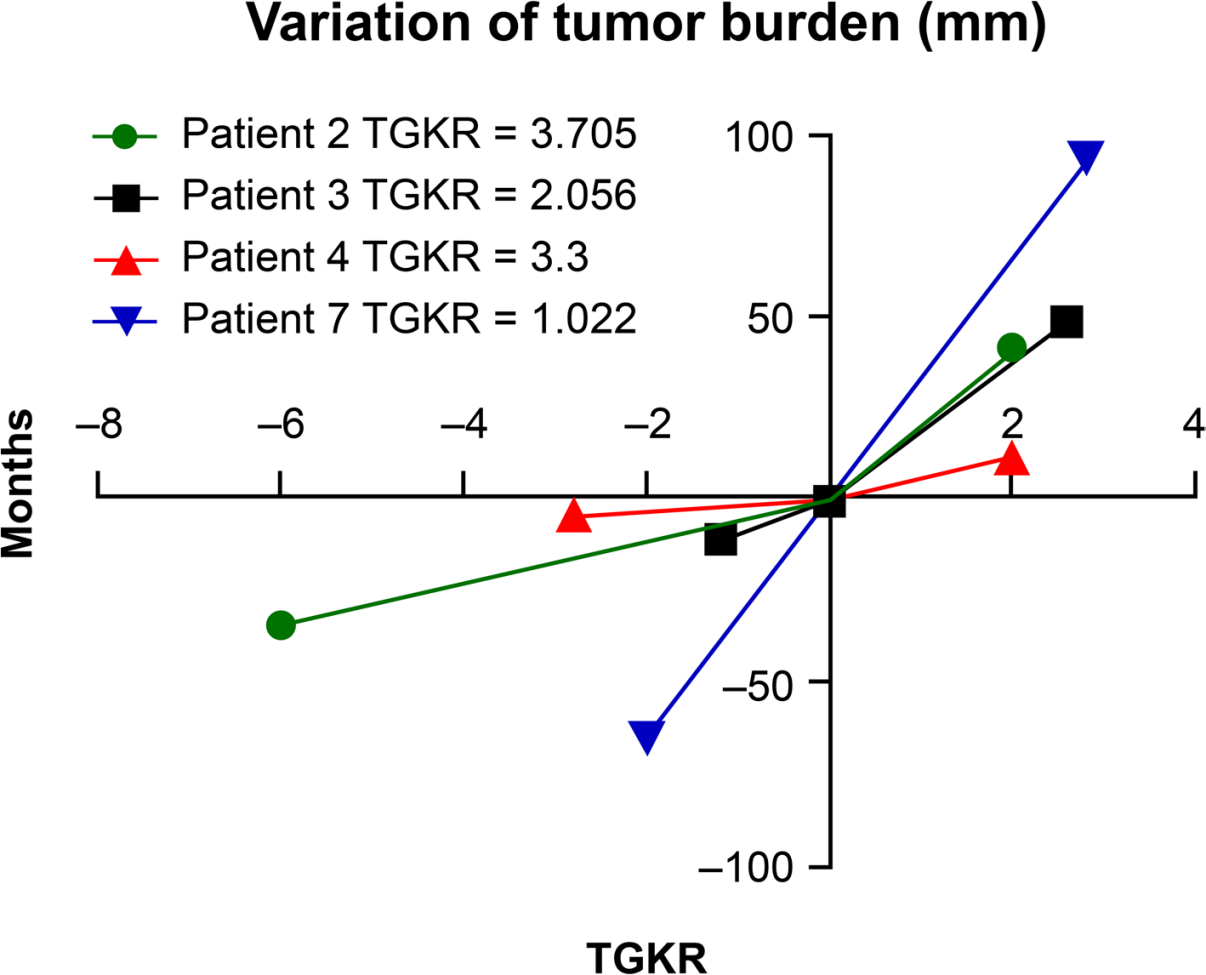
The tumor size before treatment, at baseline, and after treatment in patients with progression. The tumor growth kinetics ratio (TGKR) is also presented

Because we observed rapid disease progression in Patient No. 5 shortly after the administration of rituximab, we questioned whether B cell depletion using rituximab monotherapy could cause HPD. We used TGKR to evaluate tumor growth rates in patients with disease progression [14]. TGKR could be evaluated in three patients treated using protocol 1 (Patient No. 2–4). All three patients experienced HPD, as presented in Fig. 3. Regarding patients treated using protocol 2 who experienced disease progression (Patient No. 7–8), Patient No. 8 could not undergo CT after disease progression because superior vena cava syndrome prevented him from adopting the supine position during the examination. Therefore, we only evaluated TGFR in Patient No. 7, and no evidence of HPD was detected, as illustrated in Fig. 3.

### Immune cell evaluation

**Fig. 4:**
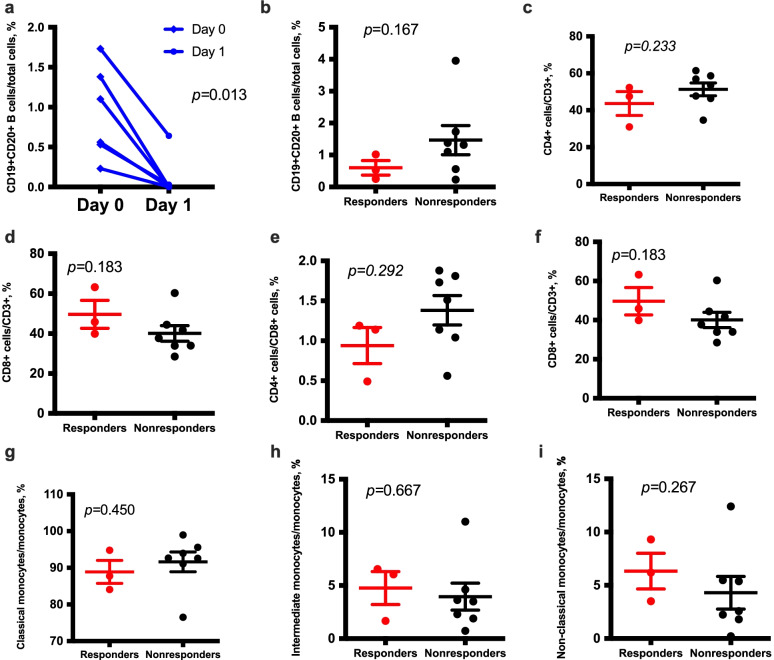
Peripheral blood immune cell profile. CD20 count on days 0 and 7. **a** Peripheral immune cell profile in non-classical monocyte responders and non-responders including **b** CD19+CD20+, **c** CD4, **d** CD8, **e** CD4/CD8 ratio, **f** regulatory T cell/CD8 ratio, **g** classical monocytes, **h** intermediate monocytes, and **i** non-classical monocytes. The results were analyzed using the Mann–Whitney U test (*n* = 3 + 7)

We investigated the changes in immune cell counts in peripheral blood. We found B cells were significantly depleted in all patients after rituximab administration (*p* = 0.013). We examined the counts of B cells (CD19+CD20+), cytotoxic T cells (CD3+CD8+), helper T cells (CD3+CD4+), regulatory T cells (CD3+CD4+CD25+CD127−), classical monocytes (CD14+CD16−), non-classical monocytes (CD14−CD16+), and intermediate monocytes (CD14+CD16+) to analyze whether the immune cell counts in peripheral blood were correlated with treatment responses. The results demonstrated that responders had a significantly higher ratio of cytotoxic T cells/regulatory T cells in peripheral blood before rituximab treatment than non-responders (*p* = 0.017) (Fig. 4). No differences were detected in the counts of other immune cells between responders and non-responders.

## Discussion

This pilot study investigated the feasibility of rituximab combined with chemotherapy in patients with R/M HNSCC. Our study featured two treatment protocols. In protocol 1, rituximab monotherapy was used in the first 2 weeks to deplete B cells, followed by chemotherapy. One patient had an unpredicted adverse event. In addition, we analyzed the outcomes of the first four patients and found that three patients had rapid disease progression after treatment. We altered the treatment regimen to co-administer rituximab and chemotherapy on the same day. Five patients were enrolled in protocol 2, and no patients reported unpredicted adverse events. Therefore, it appeared feasible and safe to simultaneously co-administer rituximab and cytotoxic agents in patients with HNSCC, but rituximab monotherapy should not be provided to these patients because of its high risks of HPD.

The function of B cells in the TME remains controversial. A meta-analysis of expression signatures from 18,000 human tumors demonstrated that the presence of plasma cells portended good survival outcomes [15]. Plasma cells release anti-tumor antibodies to induce antibody-dependent cell cytotoxicity and phagocytosis in tumor cells [16, 17]. Recent studies demonstrated that the presence of B cells within tertiary lymphoid structures was associated with the response to immune checkpoint blockade in melanoma, renal cell carcinoma, and soft tissue sarcoma [18–20]. Conversely, B cells also can promote tumorigenesis and attenuate anti-tumor immunity [21, 22]. The presence of memory B cells is a poor prognostic factor for lung squamous-cell carcinoma, gastric cancer, and colon cancer [15]. In an experimental study, B cells produced lymphotoxin and circulating immune complexes to promote carcinogenesis [23, 24]. In addition, regulatory B cells secrete various inhibitory cytokines and molecules such as IL-10 and IL-35 to suppress effector T cells and promote regular T cell formation [25, 26]. B cell depletion using anti-CD20 agents regulated the phenotype of tumor-associated macrophages and inhibited tumorigenesis and cancer growth in a mouse HNSCC model [5]. In addition, the administration of an anti-CD 20 agent before chemotherapy will improve the efficacy of chemotherapy in vivo [5]. Therefore, preclinical studies demonstrated the complex and diverse roles of B cells in tumor growth. Our clinical observation that rituximab alone caused hyperprogression of HNSCC supported the importance of B cells as anti-tumor immune cells. More specific targeting of B cell populations, such as regulatory B cells, may be necessary to apply B cell depletion in cancer treatment.

HPD is a critical issue limiting the use of checkpoint inhibitors. Many studies revealed that some patients experience accelerated disease worsening after anti-PD-1 monotherapy [27, 28]. Interestingly, chemotherapy combined with anti-PD-1 agents carried a lower risk of HPD [27, 29]. In the Keynote 048 trial, pembrolizumab monotherapy was associated with worse PFS than cetuximab/chemotherapy and pembrolizumab/chemotherapy in the first 6 months [29], although it is difficult to determine whether the accelerated growth kinetics is due to immunotherapy or just reflect the natural history of cancer. Our study also observed rapid progression in three patients treated using protocol 1. In protocol 2, the patients received rituximab and chemotherapy simultaneously, and no one developed HPD, supporting the feasibility of rituximab and chemotherapy co-administration in patients with HNSCC.

Platinum agents are important cytotoxic treatments for HNSCC. Cisplatin is the backbone of induction chemotherapy and chemoradiotherapy [30, 31], and patients with platinum resistance have poor outcomes. In prior research, immune checkpoint blockade prolonged the survival of patients who progressed on platinum treatment [32]. However, fewer than 20% of patients respond to immune checkpoint blockade. Cisplatin/gemcitabine produced a response rate of approximately 20% in R/M HNSCC. Meanwhile, the time to progression was only 4 months, and the duration was even shorter in patients who previously received chemotherapy [33]. The small sample size in our pilot study prevented us from analyzing treatment responses. However, although all six patients (including patient No. 5) treated by rituximab plus gemcitabine/cisplatin had platinum-resistant tumors, two partial responses and one complete response were observed. Additionally, these three patients had disease-free intervals of 6–7 months. In the analysis of peripheral blood cells, these three responders had a higher ratio of cytotoxic T cells to regulatory T cells than non-responders. Several studies found that cytotoxic T cells and regulatory T cells can be used to stratify the immunophenotype of head and neck cancer and predict survival [34–36]. Whether differences in the TME cause diverse outcomes of B cell depletion warrants further investigation.

## Conclusions

Treatment with rituximab alone can cause HPD in patients with HNSCC, whereas the co-administration of rituximab and cisplatin/gemcitabine is feasible and safe. However, the clear benefit of rituximab in combination with chemotherapy is unclear at this moment. Further studies are necessary to investigate the role of B cells in the TME and develop new treatment strategies.

## Data Availability

The datasets generated and analyzed in the current study are available from the corresponding author upon reasonable request.

## Abbreviations

CT: Computed tomography
EGFR: Epidermal growth factor receptor
HPD: Hyperprogressive disease
PD-1: Programmed death 1
PFS: Progression-free survival
R/M HNSCC: Recurrent or metastatic head and neck squamous-cell carcinoma
TGKRs: Tumor growth kinetics ratios
TME: Tumor microenvironment
